# Stroke Survivors Scoring Zero on the NIH Stroke Scale Score Still Exhibit Significant Motor Impairment and Functional Limitation

**DOI:** 10.1155/2014/462681

**Published:** 2014-03-17

**Authors:** Brittany Hand, Stephen J. Page, Susan White

**Affiliations:** ^1^Occupational Therapy Division, School of Health and Rehabilitation Sciences, The Ohio State University Medical Center, 453 West Tenth Avenue, Suite 406, Columbus, OH 43210, USA; ^2^School of Health and Rehabilitation Sciences, The Ohio State University Medical Center, 453 West Tenth Avenue, Suite 406, Columbus, OH 43210, USA

## Abstract

*Objective.* To determine the National Institutes of Health Stroke Scale's (NIHSS's) association with upper extremity (UE) impairment and functional outcomes. *Design.* Secondary, retrospective analysis of randomized controlled trial data. *Setting.* Not applicable. *Participants.* 146 subjects with stable, chronic stroke-induced hemiparesis. *Intervention.* The NIHSS, the UE Fugl-Meyer (FM), and the Arm Motor Ability Test (AMAT) were administered prior to their participation in a multicenter randomized controlled trial. *Main Outcome Measures.* The NIHSS, FM, and AMAT. *Results.* The association between the NIHSS and UE impairment was statistically significant (*P* = −0.204; *p* = 0.014) but explained less than 4% of the variance among UE FM scores. The association between NIHSS total score and function as measured by the AMAT was not statistically significant (*P* = −0.141; *p* = 0.089). Subjects scoring a “zero” on the NIHSS exhibited discernible UE motor deficits and varied scores on the UE FM and AMAT. *Conclusion.* While being used in stroke trials, the NIHSS may have limited ability to discriminate between treatment responses, even when only a relatively narrow array of impairment levels exists among patients. Given these findings, NIHSS use should be restricted to acute stroke studies and clinical settings with the goal of reporting stroke severity.

## 1. Introduction

Upper extremity (UE) hemiparesis remains one of the most frequent stroke-induced impairments [1] and considerably undermines performance of valued activities. Yet, despite weeks of rehabilitation, 50% of patients retain some degree of UE weakness [2] and up to seventy percent remain unable to functionally use their paretic UEs [3] in the months after stroke.

Scores on the National Institute of Health Stroke Scale [4] (NIHSS) are associated with stroke outcomes [5–7], causing the NIHSS to be recommended for determining “appropriate treatment and predicting patient outcome” [8]. However, the “functional” measures with which the NIHSS has been associated in stroke trials [7, 9, 10] (e.g., Glasgow Coma Scale; Barthel Index) do not directly assess active UE movement or functional UE activity performance. For example, the Barthel Index ascertains the level of help that a patient requires to carry out various daily activities, but not the actual level of movement that the patient exhibits or how active movements conspire to facilitate participation in valued activities. These levels of help may be related to adaptive equipment use, available care partner support, or other factors, but do not tell the user how the client has actually responded to treatment strategies or how the client's current movement status correlates with stroke severity as measured by the NIHSS. Moreover, the ongoing and erratic nature of neurological recovery during the acute phase diminishes validity of using a single measurement point to characterize recovery or outcomes during this phase. Given these shortfalls, it remains uncertain whether NIHSS score is associated with clinically significant UE motor outcomes and often-used acute stroke methodologies for answering this important question are limited.

Lesion size remains relatively constant, spontaneous recovery has ceased, and patients have usually been discharged from all treatments during the chronic recovery phase (>6 months after ictus) [11]. Concurrently, the NIHSS is being increasingly used as a terminal outcome measure in chronic stroke recovery trials [12–14], necessitating rigorous study of how well it correlates with UE impairment and functional outcomes in this phase. Thus, a straightforward and needed method of understanding the NIHSS's association with UE outcomes is to administer it to chronic stroke survivors in parallel to validated, stroke-specific UE measures. The UE section of the Fugl-Meyer (UE FM) [15] is the most established stroke UE motor measure, iteratively evaluates active, isolated movement at each paretic UE joint in a proximal to distal sequence, and is recommended for use in stroke rehabilitative trials [16, page 239]. The Arm Motor Ability Test (AMAT) [17] is also often used in stroke trials and determines paretic UE use and the extent to which UE impairments undermine performance of valued, common activities. These measures were administered concurrently with the NIHSS to a well-characterized, national cohort of 146 chronic stroke survivors with UE hemiparesis. To increase subject homogeneity and given the proliferation of UE therapy regimens targeting patients with minimal UE impairment [18–20], all of the subjects exhibited minimal UE impairment. We hypothesized that the NIHSS scores would be positively, linearly associated with UE FM and with AMAT scores. This is because, while the NIHSS has been used as an outcome measure in stroke rehabilitative trials, the current study was expected to confirm that the NIHSS and the variable of stroke severity are associated with UE recovery in neurologically stable subjects. To our knowledge, this was the first study examining the NIHSS's association with functional UE outcomes and enrolled one of most well-characterized, controlled, subject samples to examine the NIHSS's association with recovery.

## 2. Method

### 2.1. Study Design

This study was a secondary analysis of data from the Everest randomized controlled trial of implanted cortical stimulation for UE movement in chronic stroke [21]. Outcome measures had been administered before and after intervention as part of the above trial. The current study focused solely on lesion size and values obtained on the NIHSS, FM, and AMAT prior to randomization and to any interventions taking place.

### 2.2. Subjects

Subjects were recruited for the trial from the United States using several recruitment strategies, including print advertisements placed in clinics near enrolling sites, radio advertisements in the markets of enrolling sites, and print advertisements placed in national magazines whose primary subscribers were survivors of stroke. As volunteers came forward, the following screening criteria were applied. Inclusion criteria are as follows. (1) Subjects must have had an ischemic vascular lesion (i.e., stroke), as documented by computerized tomography or magnetic resonance imaging and occurring above the level of the midbrain. (2) Stroke occurred >4 months prior to study enrollment. (3) Subjects were medically and neurologically stable as determined by medical history and documented neurological examination. (4) Score on the upper extremity section of the Fugl-Meyer scale (described later) is ≥28 ≤ 50, and active extension in the affected wrist of at least 5°. (5) Age ≥ 21 years or older at time of enrollment. (6) Women of childbearing potential must have a negative serum *β*hCG pregnancy test within 2 weeks of study entry and be willing to practice adequate contraception during the study. Exclusion criteria are as follows: (1) hemorrhagic stroke; (2) >1 stroke; (3) any neurologic condition (beyond the stroke) or physical condition that impaired function of the affected arm; (4) history of seizure disorder or a spontaneous seizure that had occurred one month or longer from stroke; (5) neurological condition that would likely reduce the safety of study participation, including central nervous system vasculitis, intracranial tumor, intracranial aneurysm, multiple sclerosis or arteriovenous malformations; (6) moderate to severe hemispatial neglect and/or anosognosia involving the affected arm; (7) severe sensory deficit, including, but not limited to, a score of 2 on part 8 of the National Institutes of Health Stroke Scale (NIHSS); (8) inability to understand, cooperate, or comply with the study procedures; (9) severe spasticity, defined as an Ashworth score of 4 in any region of the affected arm; (10) change in oral spasticity medications occurring 2 weeks prior to enrollment or botulinum toxin A injections in the affected arm 4 months prior to enrollment; (11) major active psychiatric illness that may interfere with treatment; (12) untreated or inadequately treated depression defined by a score of 19 or greater (out of 63) on the 21-question version of the Beck Depression Inventory; (13) Modified Rankin score ≥4; (14) a substantial cardiopulmonary or metabolic disorder; this includes a current serum creatinine >3.0 mg/dL, a total serum bilirubin >2.0 mg/dL, or advanced chronic obstructive pulmonary disease; (15) increased risk for myocardial infarction or other major medical complications of general anesthesia or surgery; (16) terminal illness associated with survival <12 months; (17) inability to discontinue antithrombotic therapy (e.g., antiplatelet agents or anticoagulants) perioperatively for device implantation and removal; (18) introduction in the 2 months prior to enrollment of a potentially confounding central nervous system drug (e.g., amphetamines, antiepileptics, anxiolytics, and antidepressants); (19) history of spinal cord injury, traumatic brain injury, or spontaneous subdural or epidural hematoma that has resulted in a neurologic deficit; (20) current abuse of alcohol or drugs; (21) contraindication to stimulation system placement surgery; (22) contraindication to magnetic resonance imaging (e.g., implanted metallic or electrical devices); (23) nursing a child, pregnancy, or intent to become pregnant during the study; (24) participating in another trial within 30 days of enrollment in this study; (25) patient that has a condition that, in the opinion of the investigators, would interfere with study compliance or safety.

Using the aforementioned study criteria, 146 subjects were included in the current analysis (85 males; mean age of all subjects = 56.7 ± 0.9 years; mean time since stroke onset = 58.7 ± 5.3 months; 86 subjects with hemiparesis affecting their right UEs; 83 subjects with hemiparesis affecting their dominant UE's). Subjects' mean UE FM score was 37.5 ± 0.5, their mean AMAT score was 3.0 ± 0.1, and their mean NIHSS total score was 2.7 ± 0.1.

### 2.3. Instruments

The* NIHSS *[4] examines impairment in 11 domains (e.g., level of consciousness; horizontal eye movement; facial palsy; motor arm; motor leg) using either a three or four-point Likert scale applied to each item (zero is normal function; higher score is indicative of maximal impairment). Scores on each item are then summed for a single, total score, with a higher score indicative of greater deficit and stroke severity.

Stroke severity and neurologic status may be expressed in a myriad of ways in the paretic UE. Thus, we administered established, frequently used measures of both UE impairment (i.e., active movement in each UE joint) and function (i.e., intersegmental movements put together in sequential fashion to accomplish a functional goal). The measures were administered by blinded raters at participating centers at which the trial was being conducted. All raters were certified and recertified on the outcome measures every 3 months using standardized, live, and video-based interrater reliability checks at the main study center. (a) The upper extremity motor portion of the* Fugl-Meyer scale (UE FM) *[15] was used to assess upper extremity impairment. Data arise from a 3-point ordinal scale (0 = cannot perform; 1 = can partially perform; 2 = can perform fully) applied to each item, and the items are summed to provide a maximum score of 66. The UE FM's scores have been shown to offer high test-retest reliability (total = 0.98-.99; subtests = 0.87-1.00), interrater reliability, and construct validity in similar contexts to that which was tested herein (i.e., mildly impaired, chronic stroke) [22, 23]. (b) The* Arm Motor Ability Test *[24] is used to determine activity limitations while performing common, functional skills needed for independent living. The AMAT is a 13-item test in which the subject's ability to perform activities is rated according to a functional ability scale (FA) that examines paretic limb use (0 = does not perform with paretic upper extremity; 5 = does use arm at a level comparable to less affected side) and a scale in which subjects are timed on each item (time).

### 2.4. Statistical Analyses

All three outcome measures use ordinal-level scales to rank subjects' deficits, but their total scores are continuous. The nonnormal, heterogenous distributions that are commonly seen in stroke also led us to expect that data would be nonparametric. Thus, we chose Spearman's correlation coefficient to assess our primary hypothesis (correlations between the variables), given its ability to be used when variables are ordinal or continuous and its utility in determining the association between variables in with nonparametric datasets.

**Figure 1:**
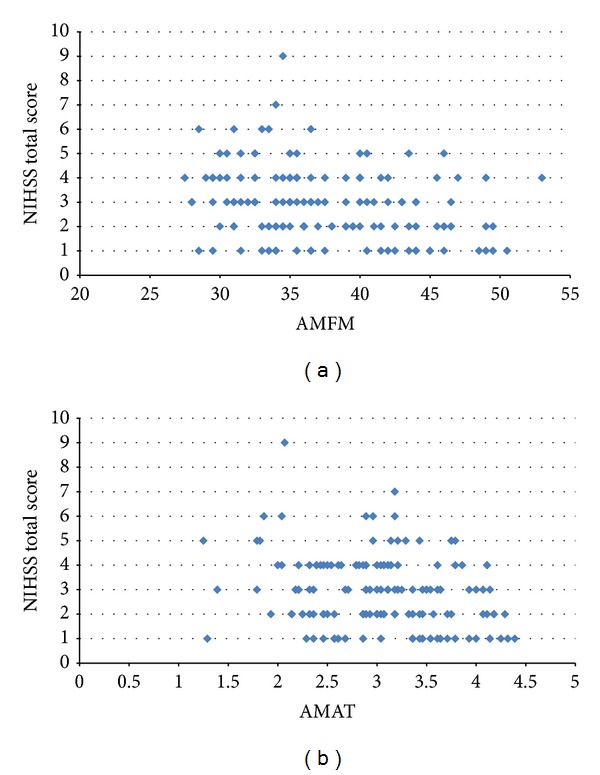
Bivariate plots comparing NIHSS scores with UE FM scores (a) and NIHSS scores with AMAT scores (b).

To corroborate findings from the above correlational analyses, several, additional subanalyses were performed. (a) First, we graphically depicted relationships between the NIHSS and the UE FM and the NIHSS and the AMAT using bivariate plots (Figures 1(a) and 1(b), resp.). In addition to the numbers derived from the correlational analyses, these plots provided a pictorial representation of correlations (or lack thereof) between the NIHSS and our two UE functional variables, allowing the reader to graphically observe the relational trends between data points on the measures and visualize if relationships existed. (b) Furthermore, after computing a median NIHSS score of 3 in our entire sample, we examined associations of UE FM and NIHSS scores among a subgroup of subjects scoring “low” on the NIHSS (NIHSS ≤ 2), and a second subanalysis examined the association between NIHSS and UE FM scores in “high” NIHSS scorers (NIHSS ≥ 3). Given the heterogeneity of stroke, we wanted to tease out data from subjects who were more impaired (NIHSS ≥ 3), as these subjects may have exhibited more variable recovery trajectories that may have deleteriously affected the overall correlational analysis. Confirming that the data trends were the same among these two groups would confirm that no such “noise” existed and provide further confirmation of the validity of our findings. (c) For the final subanalysis, we identified subjects with a NIHSS score of zero and ran a “side by side” comparison of their UE FM and AMAT scores to the UE FM and AMAT scores of subjects with nonzero NIHSS scores. A score of zero on the NIHSS is said to be indicative of no impairment. We felt that if the subjects deemed to be a “zero” on the NIHSS which exhibited similar UE motor score trends (i.e., the UE FM and AMAT) to those subjects deemed to still have motor impairment, this would further question the validity of the NIHSS's measurement capabilities.

## 3. Results

The association between the NIHSS and UE FM was statistically significant (*P* = −0.204; *p* = 0.014) but was not practically significant [25] as it explained less than 4% of the variance in the UE FM scores. The association between NIHSS total score and the AMAT was not statistically significant (*P* = −0.141; *p* = 0.089) (Table 1).

Using bivariate plots, Figure 1(a) confirms that there is significant variation in the values of UE FM for each of the observed NIHSS values. The relationship between UE FM and NIHSS was explored further by segmenting the subjects into two groups based on the median NIHSS value of 3. Subjects with NIHSS scores of 0 through 2 were assigned to a low NIHSS group (*n* = 70) and those with scores greater than or equal to 3 were assigned to a high NIHSS group (*n* = 76). The association between NHISS and UE FM for the low NIHSS subgroup was not statistically significant (*P* = 0.091, *p* > 0.05). The association between NHISS and UE FM for the high NIHSS subgroup was also not statistically significant (*P* = −0.084; *p* > 0.05).

**Figure 2:**
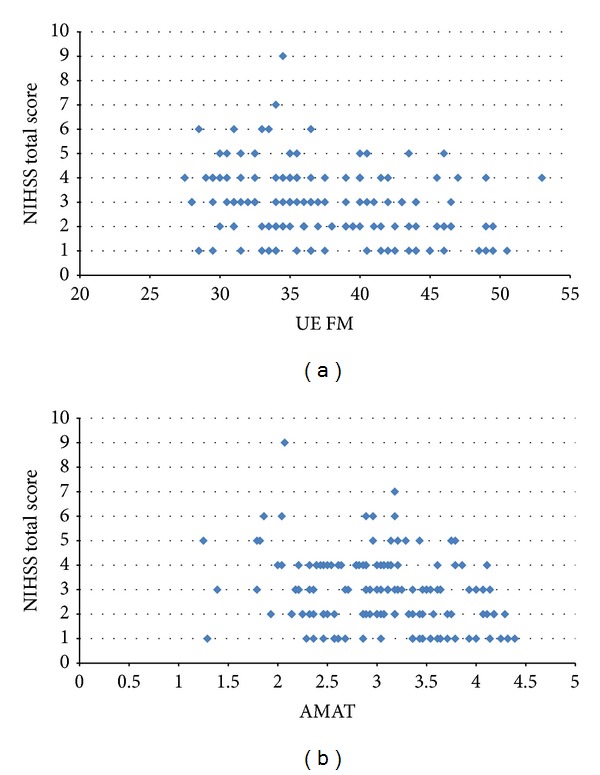
Comparison of subjects with zero and nonzero NIHSS scores on the UE FM and on the AMAT.

One final analysis was performed to confirm the relationships between NIHSS total score with UE FM and AMAT scores. This analysis focused on comparing the 12 subjects with a NIHSS score of 0 to the remaining subjects with nonzero NIHSS scores. The average UE FM and AMAT scores were not statistically different for the two subsets of subjects (Figure 2). 

## 4. Discussion

Due to growing prevalence of stroke risk factors and improved acute care methods, the number of stroke survivors exhibiting UE impairment is likely to increase. The NIHSS is commonly used to characterize neurologic status and stroke severity and reportedly predicts stroke outcomes. However, the latter finding was not derived from how well patients moved or performed valued activities in the weeks after ictus, but rather from indirect measures such as the level of assistance needed from others and/or level of consciousness. To rigorously test its relation to UE outcomes, we administered the NIHSS, the UE FM, and the AMAT to 146 stroke survivors. The outcomes were assessed during the chronic period of recovery to reduce the impact of commonly occurring, extraneous factors during the acute period such as spontaneous neurologic recovery and concurrent pharmacologic and therapy treatments.

We hypothesized that the NIHSS would be strongly associated with UE FM and AMAT scores. This hypothesis was not confirmed. We then deployed additional analyses to verify the validity of these findings and to provide insight as to why the results were negative. These analyses revealed that patients with identical NIHSS scores exhibited considerable variability on the UE FM and AMAT. Specifically, among those who received a score of one on the NIHSS the mean score was 40.06 (SD = 6.54, range: 28.5–50.5) on the UE FM and 3.27 (SD = 0.77, range: 1.29–4.39) on the AMAT. Similarly, patients scoring a “zero” on individual NIHSS items or on the entire scale, continued to exhibit UE motor deficits to varying degrees. Taken together, these findings suggest that the NIHSS may have limited ability to discriminate between a range of UE levels, even given a relatively narrow array of impairment levels as was the case in this study. This is of particular concern given that most studies do not delimit patients' movement levels as closely as we did in this study and given the heterogeneity of patients encountered in clinical practice. As a result of our findings, the NIHSS may be suboptimal for use as an outcome measure in stroke rehabilitative trials, since its use could give rise to Type II errors (i.e., not detecting a change that is actually present). This is because our data suggest that changes in function post intervention may, in some cases, not be detected using the NIHSS and/or that the variability in response to the intervention may go undetected if the NIHSS is used. Similarly, clinicians should use caution in making assertions about patients' UE rehabilitative needs or potential based on NIHSS scores.

In parallel to the adequacy of the measure, one should consider whether the evaluator is ideally qualified to systematically assess paretic UE deficits and whether raters will be able to ascertain how these deficits conspire to diminish functional performance. NIHSS training does not convey these skills, and clinicians using the NIHSS in the acute setting may not have learned this skill as part of their academic or subsequent training. Given this and our study findings, it is suggested that NIHSS administration is restricted to use in acute stroke studies and clinical settings with the goal of characterizing stroke severity.

In obtaining the above results, this study used a large study sample, and thus, a large number of observations were incorporated into the analyses. This large number of observations constitutes a study's strength, as it increases the likelihood that study findings are valid. Sample characteristics are also a study's strength, including enrollment of chronic stroke patients, which reduces the possible confounding effects of extraneous factors present during the acute recovery period. Given that the impairment group enrolled in this study is eligible for several, recently developed, rehabilitative therapies [14–16], information from this study is expected to be useful in selecting the best outcome measures for studies enrolling patients from this group.

This study used highly specific inclusion criteria regarding stroke severity and time after stroke, which may not be generalizable to all stroke patients. This constitutes a possible study limitation. However, the application of these criteria also constitutes a strength, as their use resulted in a well-defined study population of patients who were neurologically stable and were not receiving concurrent interventions. Future studies should seek to replicate these findings in acute stroke populations as well as in populations with greater stroke severity.

## 5. Conclusion

Our findings indicate that, in a large cohort of stable stroke survivors, NIHSS total scores were not associated with scores on established measures of UE impairment and function. Based on these findings, we conclude that the NIHSS should be restricted for use in acute settings to determine stroke severity and should not be used to predict or assess UE outcomes in the months and years after ictus. Given its subpar properties in mild and stable of populations, an important next step is to examine the NIHSS's measurement attributes in relation to well-established UE motor measures in more acute and neurologically unstable groups.

## Figures and Tables

**Table 1:** Correlation matrix of NIHSS, UE FM, and AMAT scores.

	NIHSS total score	UE FM	AMAT
Spearman's rho			
NIHSS total score			
Correlation coefficient	1.000	−0.204*	−0.141
Sig. (2-tailed)	.	0.014	0.089
*N*	146	146	146
UE FM			
Correlation coefficient	−0.204*	1.000	0.588**
Sig. (2-tailed)	0.014	.	0.000
*N*	146	146	146
AMAT			
Correlation coefficient	−0.141	0.588**	1.000
Sig. (2-tailed)	0.089	0.000	.
*N*	146	146	146

*. Correlation is significant at the 0.05 level (2-tailed).

**. Correlation is significant at the 0.01 level (2-tailed).

## Abbreviations

UE: Upper extremity
UE FM: Upper extremity Fugl Meyer
AMAT: Arm Motor Ability Test
NIHSS: National Institutes of Health Stroke Scale.
