# Erratum to “Stroke Survivors Scoring Zero on the NIH Stroke Scale Score Still Exhibit Significant Motor Impairment and Functional Limitation”

**DOI:** 10.1155/2014/542638

**Published:** 2014-08-12

**Authors:** Brittany Hand, Stephen J. Page, Susan White

**Affiliations:** ^1^Occupational Therapy Division, School of Health and Rehabilitation Sciences, The Ohio State University Medical Center, 453 West Tenth Avenue, Suite 406, Columbus, OH 43210, USA; ^2^School of Health and Rehabilitation Sciences, The Ohio State University Medical Center, 453 West Tenth Avenue, Suite 406, Columbus, OH 43210, USA

Figure 1 was inserted twice and Figure 2 was omitted. Below are the correct figures.

## Figures and Tables

**Figure 1:**
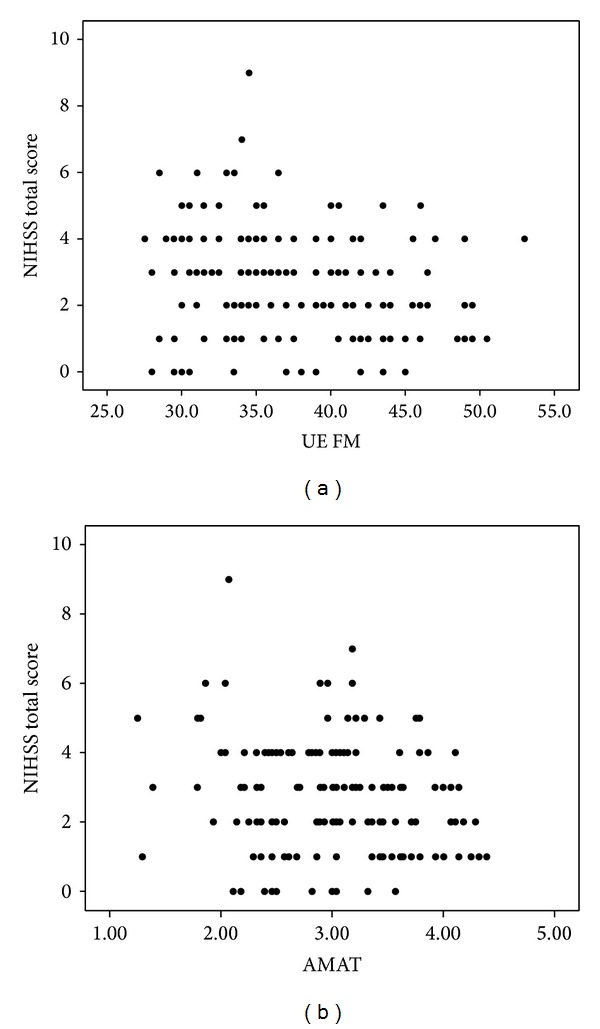
Bivariate plots comparing NIHSS scores with UE FM scores (a) and NIHSS scores with AMAT scores (b).

**Figure 2:**
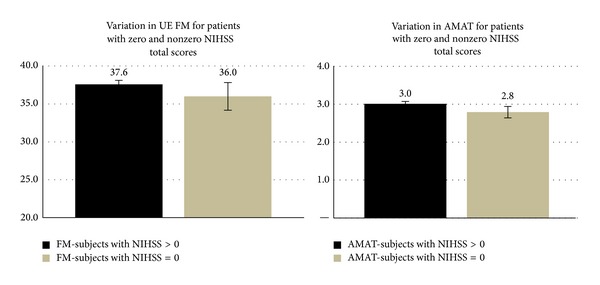
Comparison of subjects with zero and nonzero NIHSS scores on the UE FM and on the AMAT.

